# Systematic Reviews: Are They Actually Well Conducted and Reported in Accordance with PRISMA? 

**DOI:** 10.29252/beat-080110

**Published:** 2020-01

**Authors:** Masoumeh Gholizadeh, Mehrdad Amir-Behghadami, Ali Janati

**Affiliations:** 1 *Iranian Center of Excellence in Health Management (IceHM), Department of Health Service Management, School of Management and Medical Informatics, Tabriz University of Medical Sciences, Tabriz, Iran*; 2 *Student Research Committee (SRC), Tabriz University of Medical Sciences, Tabriz, Iran*

***Dear Editor,***

Recently, Ayalew *et al*. have published an article, titled, “Drug related hospital admissions; A systematic review of the recent literatures” in Bull Emerg Trauma in 2019, 7^th^ volume and 4^th^ issue that has been caught our attention [1]. Even though the results of the study are interesting, there are flaws due to the authors' negligence in the method, which leads to ambiguity in the interpretation of the findings. Therefore, the points expressed in this letter indicate what are needed to be perused in reporting systematic reviews.

Systematic reviews, widely regarded as studies with the highest level of evidence, are increasingly being used to guide policy decisions and orient future research [2]. There are several guidelines that help to maintain a level of homogeneity and quality for reporting these studies. It is suggested that systematic reviews to follow a set of strict established guidelines, such as PRISMA, Cochrane or JBI [3, 4]. Therefore, the lack of any mention to the use of these guidelines raises the question of whether such a guideline was not used or merely not mentioned, even if the study follows all the principles of the guidelines. Most researchers report their design in accordance with PRISMA because its use reduces the bias and improves the quality [3].

According to PRISMA guidelines, some items have not been well reported. Searching for information as a main component of a systematic review must be comprehensive and attempt to retrieve all of the available evidences that are potentially relevant to the subject of the study [3]. For this reason, it is essential to search multiple databases using a comprehensive search strategy, although it is time consuming [5]. Generally, a systematic review should search international databases such as PubMed, Cochrane Library, EMBASE, Scopus and Web of Science; and national databases such as Magiran, SID and BKNS in order to ensure awareness of health care practices and policies [5]. 

In addition to searching the databases, the snowballing search strategy is helpful in identifying other eligible studies according to the references lists and citation of the included studies [6]. Systematic reviews that claim to perform a comprehensive search must also attempt to search for gray literature. Gray literature covers the unlisted evidence in electronic databases, so it is recommended to search for gray literature sources in order to minimize bias in search results. Gray literature includes technical reports, official publications, conference papers, theses, patent inventions, ongoing research that is usually provided by academic, government, and professional organizations [7]. 

However, we found that the authors have searched only one database, PubMed. Searching a single database can reduce sensitivity to as low as 66% [8]. In addition, search strategies must include both keywords or free-text words and index terms that used by some important bibliographic databases to describe the content of each published article using a “controlled vocabulary” [7]. However, keyword combinations are not transparent. Also, according to the PRISMA statement, it is suggested that the search strategy to be devised at least for PubMed and replicated for the other electronic databases [3].

The inclusion and exclusion criteria could also be clear. It is not clear whether these articles are expected to meet all or any of these criteria. Although excluding non-English articles reduces the power of the article, it should be mentioned in exclusion criteria too. Depending on the subject, it is recommended to use the PICOS (Population, Intervention, Comparison, Outcomes, Study design) format. PICOS is an established framework for formulating the research questions and determining eligibility criteria for the literature search [7]. 

After removing duplicate references, the title and abstract of the retrieved studies are screened initially and subsequently full-text studies are read and then evaluated qualitatively, but the quality of the studies has not been appraised in this study. Analyzing and interpreting preliminary studies in a systematic review requires qualitative assessment and bias sensitivity assessment, because poor quality studies affect the quality of the results and distort the results of the studies [9, 10]. 

We would like to know the quality of the included studies is not assessed, an oversight or an intentional decision is present, and if so, what reason is behind it. It is recommended to use the JBI Critical Appraisal Checklist developed and approved by the JBI Scientific Committee when evaluating the quality, as they have specific checklists for a variety of studies [9]. Systematic review findings are more valid than other types of reviews, because the systematic method used searches the reduction in bias and the increase rigor in identifying and synthesizing the best evidences available for a particular question. 

Therefore, when searching for evidence, researchers must strive to retrieve all the studies eligible and consider them for inclusion in their review. We believe that these points and recommendations can improve the quality of the methodology of the study and future research. As a result, our final recommendation for more transparency is that researchers and journals adhere to use PRISMA. It is obvious that its use will improve the quality of systematic review and prevent such ambiguities. 

## Conflict of Interest:

Not to declare.
